# Liquid biopsy in NSCLC: a new challenge in radiation therapy

**DOI:** 10.37349/etat.2021.00038

**Published:** 2021-04-30

**Authors:** Annarita Perillo, Mohamed Vincenzo Agbaje Olufemi, Jacopo De Robbio, Rossella Margherita Mancuso, Anna Roscigno, Maddalena Tirozzi, Ida Rosalia Scognamiglio

**Affiliations:** Department of Advanced Biomedical Sciences, University “Federico II” School of Medicine, Via Sergio Pansini 5, 80131 Napoli, Italy; Università degli studi della Campania, Italy

**Keywords:** Lung cancer, liquid biopsy, personalized therapy, radiotherapy, tissue biopsy

## Abstract

Lung cancer is the most common cancer and the leading cause of cancer mortality worldwide. To date, tissue biopsy has been the gold standard for the diagnosis and the identification of specific molecular mutations, to guide choice of therapy. However, this procedure has several limitations. Liquid biopsy could represent a solution to the intrinsic limits of traditional biopsy. It can detect cancer markers such as circulating tumor DNA or RNA (ctDNA, ctRNA), and circulating tumor cells, in plasma, serum or other biological fluids. This procedure is minimally invasive, reproducible and can be used repeatedly. The main clinical applications of liquid biopsy in non-small cell lung cancer (NSCLC) patients are the early diagnosis, stratification of the risk of relapse, identification of mutations to guide application of targeted therapy and the evaluation of the minimum residual disease. In this review, the current role of liquid biopsy and associated markers in the management of NSCLC patients was analyzed, with emphasis on ctDNA and CTCs, and radiotherapy.

## Introduction

Lung cancer is the most common cause of cancer incidence and mortality worldwide with 2.1 million newly diagnosed cases and 1.8 million deaths in 2018 [1]. According to the World Health Organization (WHO), lung cancers are divided in two main groups: non-small cell lung cancer (NSCLC; 85% of cases) and small cell lung cancer (SCLC; 15% of cases). The predominant histological subtypes of NSCLC are adenocarcinoma (ADC; 40% of cases) and squamous cell carcinoma (SqCC; 20% of cases) [2]. The majority of NSCLC are diagnosed when the disease is too extensive to be surgically removed. For these patients, chemotherapy, radiotherapy (RT) and targeted drug therapy are the standard of care. The gold standard for stage I and II NSCLC is surgery, possibly followed by adjuvant cisplatin-based chemotherapy if histological examination shows risk factors. For patients with contraindications or who are unfit for surgery, the first choice is RT as stereotactic body irradiation (SBRT) for stage I-II N0 or as concurrent definitive chemoradiotherapy for N+. Stage III is a heterogeneous group: potentially resectable tumors (stage IIIa) should be treated with neoadjuvant chemotherapy followed by surgery and adjuvant RT whereas unresectable tumors (some stage IIIa and all stage IIIb-IIIc) should be treated with concurrent definitive chemoradiotherapy followed by durvalumab, an immune checkpoint inhibitor that has been shown to improve both progression free survival (PFS) and overall survival (PACIFIC trial). In the metastatic setting, several factors such as histology, age, performance statistics, comorbidities and molecular pathway alterations should be considered in the selection of the best treatment strategies, which may include chemotherapy, immunotherapy and targeted drug therapy. Therefore, following morphological diagnosis, it is very important to evaluate predictive biomarkers of therapy. Two testing streams have evolved, one for the detection of targetable oncogenic alterations and the other for immune-oncology related biomarkers.

Diagnosis in NSCLC patients is based on tissue biopsy. Today, it must determine not only the histological subtypes (NSCLC *vs.* SCLC or SqCC *vs.* ADC) but also the potential presence of specific molecular alterations. However, this procedure has several limitations. Patient comorbidities and/or small nodules can make obtaining biopsies difficult and risky. As reported by Overman et al. [3], the complication rate for intrathoracic solid organ biopsies was 17.1% (36 of 211 biopsies). In some cases, the samples obtained are small and the content of neoplastic cells is insufficient to identify both the histotype and molecular alterations. In others cases, the samples obtained may be completely free of neoplastic cells [4]. In addition, as demonstrated in the study of Gerlinger et al. [5], lung cancer is characterized by a remarkable level of inter-tumor and intra-tumor heterogeneity. This often makes the tissue biopsy unrepresentative of the complete genetic makeup of the neoplasia. The procedure is expensive and not easily repeatable, and, for this reason, cannot be used to evaluate the temporal evolution of the disease or response to treatments. The identification of new mutations or genetic modifications is necessary to establish the most appropriate therapy for NSCLC patients, but it is evident that serial tissue biopsies are not the most suitable tools for this purpose.

In these situations, liquid biopsy could be a turning point for patient management. It can detect cancer markers such as circulating tumor DNA or RNA (ctDNA, ctRNA), and circulating tumor cells (CTCs), in plasma, serum or other biological fluids. Liquid biopsy makes it possible to access material derived from multiple tumour deposits through a procedure which is minimally invasive and repeatable. It has various potential clinical applications, such as early detection of tumor, identification of mutations for targeted therapy, evaluation of response to treatment, early identification of resistance and rapid detection of relapses.

In this review, we will analyze the current role of liquid biopsy in the management of NSCLC. The primary focus of the paper is on liquid biopsy and associated markers, especially ctDNA and CTCs, and their potential influence on RT.

## ctDNA and RNA

ctDNA is the fraction of cell-free DNA that derives from lysis/necrosis of tumor cells, from tumor destruction by macrophages or by direct secretion [6]. Although circulating cell-free DNA (cfDNA) includes all DNA fragments present in biological fluids, including those of neoplastic origin, it mainly derives from non-malignant hematopoietic cells. cfDNA was first identified in the blood by Mandel and Metais in 1948 [7]. ctDNA levels can vary enormously over time and between patients [8] and are influenced by tumor-related factors such as volume (stage TNM) and rate of proliferation, but also by factors affecting the systemic circulation. In patients with NSCLC the ctDNA level is usually 0.02–3.2% of cfDNA [9]. cfDNA is rapidly eliminated from the blood (the half-life of these fragments typically varies from minutes to 1–2 h), and ctDNA can therefore provide real-time information on the tumor [10]. cfDNA usually has a size of 166 base pairs. This is similar to the nucleosomal fragments released during apoptosis, which could represent the primary source of cfDNA [11]. The size of ctDNA has long been debated and recent studies suggest that ctDNA is shorter than cfDNA [12–13]. ctDNA carries many of the molecular aberrations present in the tumor (such as single-nucleotide mutations [14] and methylation changes [15]) and this distinguishes it from cfDNA and permits its use as an oncological marker. It allows identification of mutations in various genes, such as *EGFR*, Kirsten rat sarcoma viral oncogene homolog (*KRAS*), *BRAF*, etc. The best source for ctDNA research is plasma since the presence of coagulation inhibitors prevents the lysis of white blood cells and therefore dilution of ctDNA by cfDNA [16]. The sample is typically acquired from peripheral veins into an tube and in order to minimize sample alteration, the time between blood sample collection, plasma isolation, ctDNA centrifugation and ctDNA extraction should be reduced as much as possible [17]. One solution to minimize the time is to perform all the phases of the analysis in the same laboratory or, alternatively, to use special tubes that prevent the release of cfDNA and the degradation of ctDNA (PAXgene Blood DNA tubes or Cell-Free DNA BCT tubes) [18, 19]. Once collected, the blood is filtered or subjected to low and high speed centrifugation cycles [20].

After extraction, ctDNA profiling can be based on analysis of single mutations [generally through polymerase chain reaction (PCR)] or more broadly, using next generation sequencing (NGS) [21]. For PCR, quantitative PCR (qPCR) analysis is based on quantitation of the intensity of fluorescent light emitted by the probes in every PCR cycle, while droplet digital PCR (ddPCR) separates the sample into droplets containing either 1 or 0 molecules of DNA for subsequent amplification. Similar to ddPCR, BEAMing is a digital PCR method which uses water droplets in an oil emulsion as reaction vessels containing a mixture of template, primers and PCR reagents, and magnetic beads. NGS can be performed using several platforms, such as Illumina (San Diego, California) or IonTorrent (ThermoFisher Scientific, Waltham, Massachusetts), that sequence nucleic acids through a large number of parallel reads and their subsequent alignment to a genomic reference standard. This technology allows to analyze whole transcriptomes or a small group of RNAs. In contrast to PCR, NGS can detect rare and previously uncharacterized alterations and is particularly useful for detection of deleterious mutations which can accumulate in diverse positions within some tumor suppressor genes. Moreover, costs and analytical time associated with NGS have reduced remarkably.

As well as ctDNA, ctRNA is the fraction of circulating cell free RNA derived from tumor cells and it is detectable in the fluids of cancer patients. Compared with ctDNA, ctRNA is more unstable and it degrades very quickly. Its half-life can be increased by association with proteins or proteolipid complexes. In the context of ctRNA, miRNAs represent a promising biomarker for NSCLC. miRNAs are small noncoding RNAs (21–25 nucleotides) which modify target gene expression post-transcriptionally via inhibition on mRNA translation and induction of mRNA degradation. miRNAs have been associated with the main hallmarks of NSCLC, including sustaining proliferative signaling (miR-7, miR-30, miR-34), evading growth suppressors (miR-641, miR-660) and activating invasion (miR-200) [22]. Recently, a panel of miRNAs has been used for diagnosis and prognostication in lung cancer [23, 24].

## CTCs

CTCs are cancer cells released from solid tumors into the blood circulation and were first observed in 1,869 by Ashworth [25]. CTCs are extremely rare (1 to 10 cells per 10 mL of blood) and have a half-life shorter than ctDNA (6–10 min for clusters *vs.* 25–30 min for single cells) [26]. Despite their short lifespan, these cells may contribute to metastasis and neoangiogenesis [27]. Several studies have shown that CTCs are an unfavorable prognostic factor in breast [28], prostate [29] and colorectal cancers [30]. Immunomagnetic separation is generally the most commonly used method for isolating CTCs. In this case, CTCs are positively enriched through the use of capture agent-labeled magnetic beads which bind to cell-surface markers [31, 32]. CellSearch assay [33], the only platform approved by the Food and Drug Administration (FDA) for CTC enumeration is based precisely on this mechanism. Conversely, it is possible to negatively enrich the CTCs by using anti-CD45 to deplete leukocytes [34]. Genomic analysis of CTCs can be performed by PCR, NGS and fluorescent *in situ* hybridization (FISH) as well as ctDNA [35].

The main advantages and limitations of ctDNA, ctRNA and CTCs detection techniques are shown in Table 1.

**Table 1.** Pros and cons of analysis methods used in liquid biopsies

**Analysis method**	**Pros**	**Cons**
qPCR	Good sensitivity and specificity Widely available	Limited to HTS and discovery studies
ddPCR	High sensitivity	Expensive
BEAming	Detection of ctDNA with very low mutant allele frequency	Expensive
NGS	High sensitivity	Bioinformatics support required
	Identification of several miRNA variants	No automation
Whole-genome sequencing	Wide application	Expensive
		Time-consuming
		Bioinformatics support required
CellSearch	FDA approved	Expensive
	Good sensitivity and reproducibility	Limited to CTCs with high EpCAM levels
		False positives due to inflammation

## Clinical applications of liquid biopsy in NSCLC

**Figure 1.**
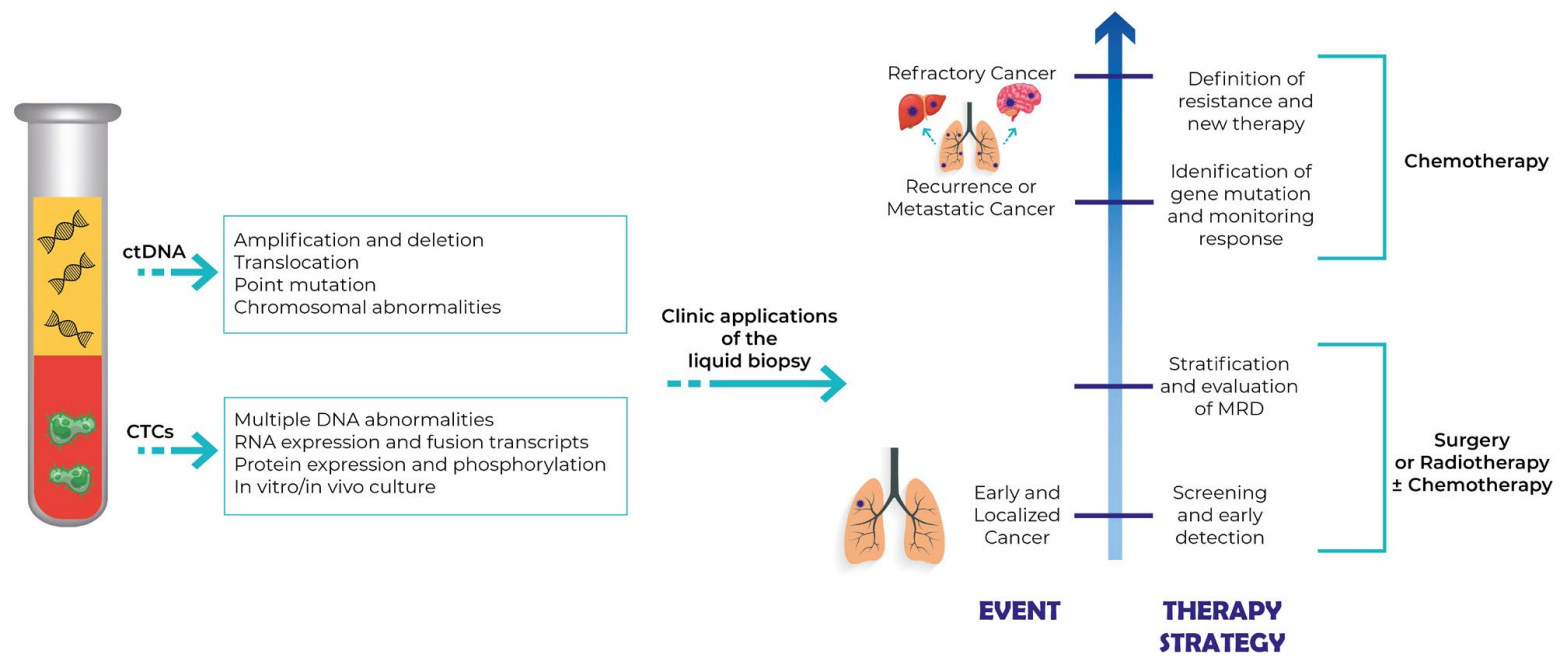
Clinical application of liquid biopsy in NSCLC

The main clinical applications of the liquid biopsy in NSCLC patients (Figure 1) are early diagnosis, stratification of risk of relapse, identification of gene mutations, and evaluation of the minimum residual disease (MRD).

## Liquid biopsy and early diagnosis in NSCLC

The rapid evolution and the lack of specific early symptoms are the basis of the late diagnosis of NSCLC and 75% of patients present with advanced stage disease at diagnosis (stage III/IV) [36]. This late diagnosis associated with reduced survival and poor prognosis as patient survival at 5 years decreases dramatically from 55.1% in stage I to 4.2% in stage IV. Early detection of lung cancer would radically change the survival of these patients. Unlike cancers such as breast, cervical and colorectal, there are no cancer screening programs for lung cancer; only in some countries is low-dose CT (LDCT) available for higher-risk individuals (55–74 years, ≥30 pack years, smoked within 15 years). The National Lung Screening Trial (NLST) study was the first to demonstrate how LDCT is associated with a 20% reduction in lung cancer mortality compared to chest X-ray [37]. Despite the advantages of LDCT, it should be remembered that this form of screening is associated with a high rate of false positives (81%) [38] and overdiagnosis of indolent tumors such as lepidic ADCs. In the NELSON trial [39] the false positive rate was reduced to 59.4% due to “nodule management protocol” (evaluation of dimension and volume doubling time), while the cumulative lung cancer detection rate was substantially higher. In addition to this imaging, liquid biopsy may be another possible tool for lung cancer screening.

In 2014 Newman et al. [40], found ctDNA in 100% of stage II–IV and 50% of stage I NSCLC patients using the Cancer Personalized Profiling by deep Sequencing (CAPP-Seq). This study has certainly underlined the possible role of ctDNA for the evaluation of the response to treatment and the level of MRD due to the link between ctDNA level and stage, but also its possible role in early diagnosis. A recent test combining the evaluation of a protein panel with mutations in the ctDNA (CancerSEEK) demonstrated a specificity > 99% and a sensitivity of 59% in 104 patients with lung cancer stage I–III [41].

Another possibility for the early diagnosis of lung cancer, offered by ctDNA, is based on the search for hypermethylation of CpG islands in the promoter regions of tumor-suppressor genes. In a Chinese study [42], the methylation frequency of 9 genes was statistically higher in the tumor tissue of subjects with NSCLC than in healthy tissue and these nine genes also showed a significantly higher frequency of tumor-specific hypermethylation in NSCLC plasma, as compared with the plasmsa fromcancer-free individuals. A smaller panel of 5 genes also showed a sensitivity and specificity of 83.64% and 74% respectively for cancer diagnosis. The genes in the panel were: *APC* (involved in cell signaling), *CDH13* (involved in cell adhesion), *KLK10* (member of the human tissue kallikrein family of secreted serine proteases), *DLEC1* (involved in cell proliferation/differentiation) and *RASSF1A* (involved in cell-cycle control). Except *KLK10*, all other genes were already known to have a role in lung cancer. The methylation status was detected using methylation-specific PCR. In Wielscher et al. [43], the methylation status of cfDNA was used as screening in subjects with lung cancer, interstitial lung disease (ILD), chronic obstructive pulmonary disease (COPD), and healthy controls. The test had 88% sensitivity and 90% specificity *vs.* the controls, and 88% *vs.* the ILD and COPD. In another study, the methylation of 3 genes was used to evaluate subjects with suspected lung nodule of these 150 had a stage I–II NSCLC and 60 were controls, the test had a sensitivity of 93% and a specificity of 62% [44]. More recently, Liang et al. [45], evaluated the role of the methylation profiling of cfDNA as a method for early detection of NSCLC and demonstrated that their method was a valid tool for differentiation between neoplastic lesions and benign pulmonary nodules.

Unlike other tumors, the detection of CTCs is complex in NSCLC. In Krebs et al. [46], CTCs were detected only in 23% patients with NSCLC stage III–IV using the CellSearch system. The combined use of different detection techniques (CellSearch and Isolation by size of epithelial tumor cells) increased detection rates [47]. Ilie et al. [48], suggested a role for CTCs in the early detection of lung cancer in subjects with COPD. In their study, 168 subjects with COPD were evaluated with annual LDCT and, in 5 of these patients, CTCs were found 1 to 4 years before the CT finding of lung nodules then recognized as tumors. CTCs were not found in the control group. In Yu et al. [49], the efficacy of folate receptor (FR)-based CTC detection method in the diagnosis of NSCLC was evaluated. This method had a sensitivity of 73.2% and a specificity of 63.2% (also in stage I). The authors stressed that their method showed greater diagnostic efficacy than other clinical markers [neuron-specific enolase (NSE), carcinoembryonic antigen (CEA), CA125, cyfra21-1, and squamous cell carcinoma antigen (SCC Ag)].

Similar to ctDNA, miRNAs can be used for early detection of NSCLC. In Ma et al. [50], analysis of two miRNAs (miRs-19b-3p and -29b-3p) identified NSCLC with a sensitivity of 72.62% and a specificity of 82.61%. These values increased in the identification of the SqCC (80% sensitivity and 89.86% specificity). Moreover, the panel formed by miR-193b, miR-301, miR-141 and miR-200b [51] showed great accuracy in discriminating patients with NSCLC (AUC 0.985). In the study of Dou et al. [52], 480 patients were analyzed (120 NSCLC and 360 healthy controls). The authors showed that the expression of let-7c and miR-152 in plasma were downregulated in NSCLC patients. These biomarkers also correlated with histological classifications, differentiation status, lymph node metastasis and stage classifications so they could represent a tool for identify patients with NSCLC.

In the future, combined use of LDCT and introduction of liquid biopsy into clinical practice may allow earlier diagnosis by increasing the number of patients susceptible to local radical treatments such as surgery and SBRT [53]. However, to date, the lack of appropriate technologies and standardized protocols does not make it possible to use liquid biopsy in daily clinical practice.

## Liquid biopsy and stratification of the risk of relapse in NSCLC

To date, modern RT techniques such as intensity modulation radiotherapy (IMRT) are highly conformational so this system can deliver high doses to the target volume while preserving the surrounding healthy tissues [54]. Different biological parameters influence cancer cells’ sensibility to damage induced by ionizing radiation (IR), such as hypoxia, reoxygenation and cellular repopulation capacity, damage repair ability, cellular redistribution of cells in the different phases of cell cycle, as well as the intrinsic radiosensitivity of each type of tumor [55, 56]. These radiobiological features explain both the heterogeneity of the response to the RT of the different tumors or the same tumor in different patients, and individual variety in the development of adverse events by RT. The tumor genome evolves dynamically over time and accumulates genetic alterations in different subclones [57]. This feature results in an intratumoral heterogeneity both spatially (i.e. between the primary tumor and the metastatic sites or, even, within the tumor nodule itself) and temporal (i.e. the bio-molecular characteristics of the tumor may change over time). Blood ctDNA levels change continuously according to tumor size, cancer stage, treatment status, etc. In this way, liquid biopsy could be a useful tool to quantify ctDNA serially to obtain a dynamic picture of the molecular evolution of the disease. It could play a very promising role in RT to guide the decision-making process for the treatment strategies and allowing monitoring of the response to the treatment, resistance development and disease progression.

## ctDNA before RT: radiosensitivity and radioresistance

Tumor genetic evaluation has become increasingly important in clinical practice, in particular to guide targeted therapies [58], but also has a potentially crucial role in RT. The discovery of prognostic and predictive biomarkers could aid stratification of patients based on the probability of responding to treatment or development of adverse events [59, 60]. In the future, such stratification will help in the choice between intensification or de-escalation dose treatments [61] and, therefore, to the development of increasingly personalized RT.

Quantitative analysis of pre-treatment ctDNA concentration in plasma is useful in the prognostic evaluation, in the follow-up [62–65] and in the identification of specific genetic alterations associated with radiosensitisation. Pre-RT genetic analysis could be fundamental in the stratification of patient as potential responders and non-responders. The mutations of several genes in different tumor settings such as *NRF2* (nuclear factor erythroid 2-related factor 2), *KEAP1* (kelch-like ECH-associated protein 1), and *KRAS*, have been identified as radiosensitising or radioresistant factors [66, 67]. One of the most promising biomarkers is the mutation of the KEAP1-NRF2 pathway which has been found in several cancers including NSCLC [68–72] and, in particular, in about one third of patients with SCLC. To date, the KEAP1-NRF2 pathway is known as a defense mechanism that cells activate in response to oxidative stress and to damage produced by toxins and xenobiotics with which they come into contact. This pathway is altered in cancer cells and this contributes to the development and progression of lung cancer [73, 74], and resistance to RT. In normal cells, KEAP1 binds NRF2 and targets it for proteasomal degradation [75, 76]. However, in response to oxidative stress, NRF2 is released from KEAP1, allowing it to accumulation and movs to the nucleus where it promotes the transcription of genes involved in defense against reactive oxygen species (ROS). Jeong et al. [67] found that the deletion of the *KEAP1* promoter results in constitutive activation of *NRF2* which favors tumor aggressiveness and metastasis, and upregulates ROS scavenging capacity and therefore resistance to oxidative stress. It is known that IR kills cancer cells due to double-stranded DNA damage induced by ROS [77] and the *KEAP1* deletion (and consequent activation of NRF2) therefore leads to ROS suppression. This event promotes radioresistance and limits the effectiveness of RT in NSCLC patients. Furthermore, *KEAP1*/*NRF2* mutations in patients with NSCLC seems to correlate with an increase in local recurrence after RT [67] suggesting that *KEAP1*/NRF2 status is predictive for local recurrence after RT in patients with lung cancer. This study shows that the identification of *KEAP1*/*NRF2* mutations status, assessed with the analysis of ctDNA, is an important biomarker of effectiveness of treatment [67]. It could have a predictive value in RT outcome of NSCLC patients.

Even the methylation status of the tumor could be a valid biomarker assessed with ctDNA analysis. According to several studies [78–83], methylation is correlated with the aggressiveness of the tumor and it promotes tumor progression by silencing genes involved in regulating tumor growth and metastatic potential. It has been shown that the reduction of ten-eleven translocation (TET), enzyme activity induced by tumor hypoxia, causes DNA hypermethylation [84] and hypoxia is known to be one of the main biological factors of radioresistance. Therefore, variations within the tumor microenvironment can influence the response to RT and their identification through ctDNA could be a starting point for future studies.

The role of transforming growth factor β (TGF-β) is important as it is activated in many solid tumors and is associated with malignant progression through different interactions in the tumor cells and in the surrounding microenvironment. *TGF-β* plays an important role as a tumor suppressor until invasive cancer development occurs, when a “switch” is activated, leading TGF-β signaling to promote the proliferation and invasion of cancer cells. IR can activate the TGF-β pathway with cross-talk activation with cox-2 [85] and TGF-β also plays an important role in the response of normal tissues to RT, especially in the lung [86]. ROS produced by IR promote the secretion of TGF-β [87] and its transcription increases within hours after irradiation [88]. Anti-TGF-β-antibodies have been shown to reduce inflammation, activation and expression of TGF-β and radiation-induced fibrosis [89] so inhibition of the TGF-β pathway could be very important for reducing damage, induced by irradiation, especially in lung. In addition, as demonstrated in several studies [90, 91], TGF-β promotes the survival of cancer cells after irradiation and its inhibition leads to radiosensitization in different types of cancer. Inhibition of *TGF-β* has a double role: protection of normal tissues and radiosensitization in the tumors. For this reason, studies that evaluate the inhibition of this pathway during or after treatment (conventional chemoradiation, radiosurgery) in NSCLC are very interesting.

Two important cellular targets in NSCLC are *EGFR* and *K-RAS*, whose mutations are mutually exclusive.

Usually, *EGFR* is mutated in ADC that most frequently occurs in young, Asian, female and non-smoking patients. *EGFR* is part of the family of HERs. It contains a C-terminus intracellular region that possesses the kinase activity and an N-terminus extracellular ligand-binding site; it is implicated in epithelial tissues maintenance and growth. Increased *EGFR* expression has been associated with radioresistance [92]. *K-RAS* is one of the most commonly mutated proto-oncogenes in various tumors, including in lung cancer in smokers. The activation of *K-RAS* seems to increase radiosensitivity in cell lines [93]. The role of *EGFR* and *K-RAS* in RT is still subject of numerous studies while their primary role is clear in the target therapy of lung cancer patients.

## ctDNA during RT: assessment of response to the treatment and prediction of toxicity

The half-life of ctDNA in the blood is very short so it provides a near “real-time” indicator of tumor kinetics [94–96]. The longitudinal determination of the ctDNA may reflect the rapid death of the cancer cells. Its determination during RT could be indicate the cytotoxic action of IR and thus be a marker of effectiveness and response to RT [97–99].

Another clinical application of ctDNA is the early determination of treatment resistance. It has long been known that there is (in addition to the inter-tumor heterogeneity in the response to radiation [100–102]) intra-tumor heterogeneity [103–106] and this could contribute to radioresistance. Radioresistance-promoting mutations seem to involve genes implicated in cell survival, tumor suppression, ROS, cell cycle checkpoints, etc [107]. The identification of resistant clones with ctDNA analysis could be very useful for dose modifications during RT.

The normal tissues surrounding the tumor inevitably receive a small dose of radiation which can cause the onset of acute (during treatment and in the first weeks after RT) or late (several months or years after treatment) toxicity. Several studies have evaluated the influence of genetic markers, especially single nucleotide polymorphisms (SNPs), on radiological toxicity [108]. These studies focused mainly on the genes involved in repairing DNA damage (e.g., *BRCA1*, *BRCA2*, and *ATM*), cytokines (e.g., TNF and TGFB1) and antioxidant enzymes (e.g., superoxide dismutase) [109–111], and demonstrated a potential association between SNPs in these genes and RT toxicity [112–114]. Further studies are needed to evaluate the correlation between genetic alterations and adverse events from RT in order to develop a personalized RT.

## Liquid biopsy and identification of gene mutation in NSCLC

Liquid biopsy can be used to detect mutations of genes involved in development of cancer, progression and response to therapy.

One of the most important markers in NSCLC is *EGFR*. This gene may exhibit different mutations which can influence sensitivity or resistance to tyrosine kinase inhibitors (TKIs) such as gefintib, erlotinib and afatinib. The most common mutations are sensitizing and typically consist of deletions in exon 19 and points mutation in exon 21 (L858R) [115]. The first occurs in 90% of cases while the second is less common; these mutations are associated with better response rates and longer progression-free survival. In contrast, most of the exon 20 insertions are usually associated with TKIs resistance. In addition, some patients with mutated *EGFR*, can acquire resistance to first- (erlotinib, gefitinib) or second- (afatinib) generation TKIs through the T790M mutation [116]. Prediction of the treatment outcome requires evaluation of the levels of both sensitizing and de-sensitizing mutations. The FDA approved the detection of these mutations using liquid biopsy. In a recent study, Mayo-de-las-Casas et al. [117], showed the absence of differences between the PFS of mutated *EGFR* patients detected by liquid biopsy compared to those detected by tissue biopsy after therapy with TKIs. In addition, Buder et al. [118], have highlighted how detection of T790M mutations in cfDNA can be a valuable tool to identify patients with resistance to osimertinib. It should also be remembered that detection of mutated *EGFR* in the plasma correlates with cancer responses to treatments measured with response evaluation criteria in solid tumors (RECIST) criteria [119]. Another great possibility provided by liquid biopsy is the early evidence of disease progression. Monitoring for increases in abundance of *EGFR* mutations detected in liquid biopsies (for example, an increase of 20% compared to the lowest value achieved during treatment) could predict progression 8 months before it was objectively detectable [120].

Another gene of particular importance in the NSCLC is anaplastic lymphoma kinase (*ALK*) which is translocated in 3–7% of lung cancer cases [121]. The most common fusion partner of *ALK* is echinoderm microtubule-associated protein-like 4 (EML4). Translocation can be detected by liquid biopsy using PCR or NGS and it can be helpful in guiding an optimal choice of a TKI. Recent evidence suggests that L1196M and S1206Y mutations of *ALK* cause resistance to crizotinib but not to ceritinib while I1171T and V1180L mutations cause resistance to alectinib and crizotinib, but not to ceritinib [122, 123]. Over time, several other fusion partners for which the clinical implication is not yet known, have been identified. The use of liquid biopsy may not be the most effective method to identify these new translocations.

Similar to *ALK* mutations (although less frequent; 1–3%), *ROS-1* mutations are also associated with altered sensitivity to TKIs. *ROS-1* mutations are found mainly in never smokers, ADC and younger patients. Following the results of Shaw et al. [124], crizotinib was approved by the FDA for treatment of advanced *ROS-1*-rearranged NSCLC. *ROS-1* rearrangements can also promote resistance to crizotinib through acquisition of additional mutations in the kinase domain or by “off target” alterations in parallel pathways. The G2032R mutation is the most frequent *ROS-1* resistance mutation and to date, only cabozantinib has shown activity against tumor clones carrying this mutation. Unfortunately, its toxicity limits its use in clinical practice [125].

To date, there are no specific drugs targeted against *ROS-1* mutations, as in the case of *KRAS*. However, assessment is relevant as it is associated with a worse prognosis and reduced responsiveness to EGFR-targeted TKIs [126].

Analysis of CTCs has great, but still unrealized, promise for guiding therapy decisions. To date, the only FDA-approved method for their analysis is ineffective in patients with NSCLC. In a recent study, the use of an alternative method for CTC detection has been shown to be more effective. The study, in addition to evaluating this new method, also analyzed the expression levels of PD-L1^+^, highlighting how their increase in the CTCs is associated with a higher probability of resistance to PD-1/PD-L1 inhibitors [127].

## Liquid biopsy and the evaluation of the MRD in NSCLC

To date, the follow-up of NSCLC patients is dependent on imaging which, despite improvements, it appears unable to identify microscopic disease. Another limitation of radiological methods is the difficulty of distinguishing between residual/recurrence disease and RT alterations. Today, RT is increasingly used in lung cancer thanks to the improvements of stereotactic and hypofractional methods, so it is becoming increasingly urgent to add to radiology other more sensitive methods in the search for residual disease. One of the most interesting potential uses of analysis of ctDNA is its possible role in detection of minimal tumor residual disease. MRD usually shows the presence of a small number of malignant cells in a patient otherwise considered in clinical remission. Since cancer often relapses after or during treatment, disease monitoring and treatment evaluation are important for clinicians to determine other treatment protocols. Preliminary data on the clinical utility of ctDNA in MRD detection is promising as demonstrated in several studies.

Diehl et al. [128], analysed a cohort of patients with surgically-treated colorectal cancer. They monitored ctDNA and CEA levels after tumor resection and saw that ctDNA detection was strongly correlated to an increased rate of relapse (PFS at 3 years was 90% in patients with undetectable levels of ctDNA *vs*. 0% in patients with detectable ctDNA). In addition, Bettegowda et al. [129], showed that the detection rates of ctDNA among the patients with cancer stage I, II, III, and IV were 47%, 55%, 69%, and 82%, indicating that ctDNA levels increase with cancer progression.

Regarding NSCLC, Guo et al. [130], investigated changes in ctDNA levels after surgical tumor resection in 41 NSCLC patients. They isolated ctDNA between 13 and 0 days before surgery and between 2 and 10 days after surgery, and the collected material was then compared with that isolated during surgery. Somatic driver mutations in tumor DNA (tDNA) and pre- and post-surgery plasma ctDNA sample pairs were identified by targeted sequencing in several genes including *EGFR*, *KRAS*, and *TP53*. The frequency of 91.7% of ctDNA mutations decreased after surgery. The agreement between ctDNA in plasma and tDNA was 78.1%, and the test showed a sensitivity of 69.2% and a specificity of 93.3%. Furthermore, the presence of ctDNA had a higher positive predictive value (94.7%) than that of six tumor biomarkers in current clinical use.

In Abbosh et al. [131], the first 100 participants of TRACERx trials were enrolled, and their ctDNA was analysed using tumour-specific phylogenetic approach. They identified specific single nucleotide variants (SNVs) as independent predictors of ctDNA release. In particular, in patient CRUK0013, affected by early-stage lung ADC, ctDNA was detectable after surgery but not after adjuvant chemoradiation. ctDNA remained undetectable on surveillance and this correlated with long-term disease-free survival. The authors demonstrated that ctDNA can be cleared after effective adjuvant treatment, and that ctDNA clearance corresponds with improved patient survival. The use of an ultra-sensitive method in quantizing ctDNA levels has shown that its levels correlate with tumour volume measured at CT and PET in pre-treatment plasma. Patient P13 (stage IIB NSCLC) after RT presented at imaging a massive mass that could be interpreted as a residue of disease but at the same time the ctDNA levels were undetectable. The patient stayed free from disease for 22 months evidencing the validity of the result of ctDNA. In contrast, patient P14 (stage IIIB NSCLC) presented a complete response to radiological follow-up after chemoradiotherapy but increasing levels of ctDNA. Indeed, clinical progression was demonstrated 7 months later.

CTCs can be another marker of residual disease. In a recent Spanish study [132], CTCs were detected before and 1 month after surgery in a group of NSCLC patients (stage I-IIIA). The discovery of these cells after surgery was associated with a faster relapse. In addition, the preoperative standard uptake value (SUV)_max_ value of the primary tumor was associated with postoperative CTCs presence.

As shown by the aforementioned studies, ctDNA can be used as a predictive marker for prognosis and treatment response. Their use in the evaluation of MRD could allow a greater customization of treatment. Adjuvant or salvage RT could be used in patients with increasing levels of ctDNA in the absence of clinically or radiologically evident residues while observation alone may be sufficient in patients with undetectable ctDNA levels. This approach would certainly improve the toxicity profile. ctDNA analysis may enable detection or therapy-resistant or dormant clones. It is currently impossible to obtain all this information, but ctDNA analysis may allow this in the not too distant future.

## Conclusions

Liquid biopsy is a minimally invasive method to monitor lung cancer dynamics and has a promising role in RT with an amazing impact on daily practice. Its possible uses are numerous, from early diagnosis, to guiding therapy and evaluation of residual disease.

The identification of radiosensitivity and radioresistance markers would allow a better selection of patients to undergo RT; serial monitoring of ctDNA during treatment could identify early non-responders and thus allow adjustment to the radiation dose (i.e. dose escalation or de-escalation). All this will lead to increasingly personalized therapy for individual patients. Liquid biopsy also has a role in follow-up because some biomarkers can reveal recurrence of disease well before traditional imaging techniques.

To date, the main indication to the use of the liquid biopsy is the identification of mutations to guide targeted therapy in patients without macroscopic disease, as approved by the European Medicines Agency and the FDA. Many potential applications of liquid biopsy remain unexplored and numerous studies will be needed to prove the effectiveness of this method and its inclusion in the management of the NSCLC. Despite these limitations, if the current evidence is confirmed, liquid biopsy will significantly change the therapeutic possibilities and therefore the prognosis of NSCLC patients especially in the RT field.

## Abbreviations

ADC: adenocarcinoma
*ALK*: anaplastic lymphoma kinase
CEA: carcinoembryonic antigen
cfDNA: cell-free DNA
COPD: chronic obstructive pulmonary disease
CTCs: circulating tumor cells
ctDNA: circulating tumor DNA
ctRNA: circulating tumor RNA
ddPCR: droplet digital polymerase chain reaction
ILD: interstitial lung disease
IR: ionizing radiation
KEAP1: kelch-like ECH-associated protein 1
KRAS: Kirsten rat sarcoma viral oncogene homolog
LDCT: low-dose CT
MRD: minimum residual disease
NGS: next generation sequencing
NRF2: Nuclear factor erythroid 2-related factor 2
NSCLC: non-small cell lung cancer
NSE: neuron-specific enolase
PCR: polymerase chain reaction
PFS: progression free survival
qPCR: quantitative polymerase chain reaction
ROS: reactive oxygen species
RT: radiotherapy
SCLC: small cell lung cancer
SNPs: single nucleotide polymorphisms
SqCC: squamous cell carcinoma
TGF-β: transforming growth factor β
TKIs: tyrosine kinase inhibitors
SBRT: stereotactic body irradiation
tDNA: tumor DNA

## Declarations

### Copyright

© The Author(s) 2021.
